# Development, Characterization, Optimization, and In Vivo Evaluation of Methacrylic Acid–Ethyl Acrylate Copolymer Nanoparticles Loaded with Glibenclamide in Diabetic Rats for Oral Administration

**DOI:** 10.3390/pharmaceutics13122023

**Published:** 2021-11-27

**Authors:** Omar Rodrigo Guadarrama-Escobar, Ivonne Sánchez-Vázquez, Pablo Serrano-Castañeda, German Alberto Chamorro-Cevallos, Isabel Marlen Rodríguez-Cruz, Adalí Yisell Sánchez-Padrón, Ericka Anguiano-Almazán, Ma. Concepción Peña-Juárez, Abraham Méndez-Albores, Clara Luisa Domínguez-Delgado, Crisóforo Mercado-Márquez, Betsabé Rodríguez-Pérez, José Juan Escobar-Chávez

**Affiliations:** 1 Escuela Nacional de Ciencias Biológicas Instituto Politécnico Nacional, Prolongación de Carpio y Plan de Ayala s/n. Col. Santo Tomás, Alcaldía Miguel Hidalgo, Ciudad de México 11340, Mexico; oguadarramae1400@alumno.ipn.mx; 2 Unidad de Investigación Multidisciplinaria-L 12, Facultad de Estudios Superiores Cuautitlán, Universidad Nacional Autónoma de México, Carretera Cuautitlán-Teoloyucan, Km 2.5 San Sebastián Xhala, Cuautitlán Izcalli 54714, Mexico; orzoket@gmail.com (I.S.-V.); pabloqfb@hotmail.com (P.S.-C.); adali_padron14@hotmail.com (A.Y.S.-P.); eri.qa.30@hotmail.com (E.A.-A.); maconcepcionpenajuarez@gmail.com (M.C.P.-J.); clara_ldd@yahoo.com.mx (C.L.D.-D.); 3 Departamento de Farmacia, Escuela Nacional de Ciencias Biológicas-Instituto Politécnico Nacional, Ave. Wilfrido Massieu s/n, Unidad Adolfo López Mateos, Alcaldía Gustavo A. Madero, Ciudad de México 07738, Mexico; gchamcev@yahoo.com.mx; 4 Unidad de Enseñanza e Investigación, Hospital Regional de Alta Especialidad de Zumpango, Carretera Zumpango-Jilotzingo # 400. Barrio de Santiago, 2a Sección, Zumpango 55600, Mexico; isabelmarlen05@gmail.com; 5 Unidad de Investigación Multidisciplinaria L14-A1 (Ciencia y Tecnología de Materiales), Facultad de Estudios Superiores Cuautitlán, Universidad Nacional Autónoma de México, Mexico City 54714, Mexico; albores@unam.mx; 6 Unidad de Aislamiento y Bioterio, Facultad de Estudios Superiores Cuautitlán, Universidad Nacional Autónoma de México, Cuautitlán Izcalli 54714, Mexico; crisoforo@gmail.com; 7 Unidad de Investigación Multidisciplinaria L 6 (Laboratorio de Servicios de Análisis de Propóleos), Facultad de Estudios Superiores Cuautitlán, Universidad Nacional Autónoma de México, Cuautitlán Izcalli 54714, Mexico; berope380@hotmail.com

**Keywords:** methacrylic acid–ethyl acrylate copolymer nanoparticles, polymeric nanoparticles, glibenclamide, polioxyl 40 hydrogenated castor oil, diabetes mellitus, glucose control

## Abstract

The methacrylic acid–ethyl acrylate copolymer nanoparticles were prepared using the solvent displacement method. The independent variables were the drug/polymer ratio, surfactant concentration, Polioxyl 40 hydrogenated castor oil, the added water volume, time, and stirring speed, while size, PDI, zeta potential, and encapsulation efficiency were the response variables analyzed. A design of screening experiments was carried out to subsequently perform the optimization of the nanoparticle preparation process. The optimal formulation was characterized through the dependent variables size, PDI, zeta potential, encapsulation efficiency and drug release profiles. In vivo tests were performed in Wistar rats previously induced with diabetes by administration of streptozotocin. Once hyperglycemia was determined in rats, a suspension of nanoparticles loaded with glibenclamide was administered to them while the other group was administered with tablets of glibenclamide. The optimal nanoparticle formulation obtained a size of 18.98 +/− 9.14 nm with a PDI of 0.37085 +/− 0.014 and a zeta potential of −13.7125 +/− 1.82 mV; the encapsulation efficiency was of 44.5%. The in vivo model demonstrated a significant effect (*p* < 0.05) between the group administered with nanoparticles loaded with glibenclamide and the group administered with tablets compared to the group of untreated individuals.

## 1. Introduction

Diabetes mellitus (DM) is a metabolic disorder characterized by the presence of chronic hyperglycemia that is accompanied, to a greater or lesser extent, by the alterations in carbohydrate metabolism. The origin and etiology of DM is multifactorial, but inexorably entails the existence of alterations in insulin secretion, sensitivity to action to hormone, or both at the same point in natural history [1].

Type 2 diabetes mellitus (DM2) is considered one of the non-communicable chronic diseases with the greatest impact on the quality of life of the world population and constitutes a real health problem. It belongs to the group of diseases that, due to their multiorgan complications, produces physical disability, with a considerable increase in recent years in morbidity and mortality regardless of social, cultural, and economic circumstances [2].

Many anti-diabetic drugs with different mechanisms of action are now available to treat DM2. Sulfonylureas have been used extensively for treatment of DM2 since discovered in 1942. Glibenclamide is a potential second-generation oral sulfonylurea agent that promotes insulin release to keep glucose levels in check. For this reason, it is widely used for the treatment of non-insulin dependent diabetes mellitus. The hypoglycemic action of this drug depends on the existence of a functioning number of beta cells of the islets of Langerhans of the pancreas, whose direct cytotropic effect causes the degranulation of these cells, causing a greater insulin secretion. The mechanism of action of glibenclamide seems to be initiated by the linkage of drug molecules with surface receptor in the beta cells’ surface and subsequent reduction in conductance of ATP-sensitive K+ channels [3,4,5,6].

Tablets are the only presentation of glibenclamide. The enteral route of administration is the highly preferable one due to its non-invasive nature. However, it reduces the bioavailability of the drug as the drug undergoes first-pass metabolism and incomplete drug absorption. Some disadvantages are present in the oral administration of glibenclamide highlighting: hyperglycemia can reduce the absorption of glibenclamide as it impairs intestinal motility, the dosage should be increased every two weeks for a great glycemic control, higher doses rarely further improve glycemic control and should be avoided, genetic differences can also change the response to glibenclamide (alter the effectiveness of sulfonylureas) and, the most common side effect is hypoglycemia, usually due to an excessive dosage. It is important to remember that hypoglycemia may persist for many hours and require in-hospital treatment [6,7,8,9,10].

Chemical engineering and the pharmaceutical industry have emphasized the development of such encapsulated products to improve selectivity and minimize the adverse side effects associated with many drugs. Nanotechnology has found applications in various fields such as medicine, cosmetics, environmental, and nutraceutical research areas [10]. Nanoencapsulation of drugs with biocompatible polymers can bring the advantages of nanotechnology to pharmaceutical products. International attention is increasingly being given to the use of drug delivery systems containing acrylic polymers, since these polymers are essentially insoluble in gastric liquids and may be used to impact enteric solubility characteristics to the encapsulated drug [11,12]. 

The use of nanoparticles (NPs) is a constant field expansion and plays a key role in various areas; recently, varied biomedical applications in bioimaging, drug administration and diagnosis have found. In the pharmaceutical area, it has been considered that the use of self-assembled colloidal particles and microcapsules shows great promise. The nanoparticle properties depend on their physical, chemical, or morphological characteristics [13].

After oral administration of glibenclamide, 30% of the dose is lost in the stomach; of the 70% that manages to pass to the intestine, 90% is bound to proteins, leaving only 10% as a free drug to exert its effect. This is why we have explored novel solutions to overcome the critical problems of inadequate oral bioavailability associated with poorly water-soluble medications and thus minimize the adverse side effects associated with dose. 

The main goal of this study was to encapsulate glibenclamide in nanoparticles of a methyl acrylate methacrylic acid copolymer whose characteristic is to be sensitive to pH, releasing the drug only at alkaline pH, thus reducing the loss of drug due to the action of the pH of the stomach and allowing the release in the intestine, improving drug absorption.

The main objective of this project was the development, characterization, optimization, and in vivo evaluation of nanoparticles of a methacrylic acid–ethyl acrylate copolymer loaded with glibenclamide in diabetic rats as a novel alternative treatment against type 2 diabetes mellitus. For this purpose, we prepared different nanoparticle (NP) suspensions where measurements for the size, zeta potential, polydispersity index (PDI), encapsulation efficiency were carried out and the morphology with scanning electron microscopy and the release profiles were analyzed. Therefore, the study led us to induce experimental diabetes mellitus in rats to measure the effect of orally administered glibenclamide methacrylic acrylate copolymer NPs.

## 2. Materials and Methods

### 2.1. Reagents

Glibenclamide (CPC), Kolliphor^®^ RH-40 (BASF, Mexico city, Mexico), Kollicoat^®^ MAE 100P (BASF México), distilled water, Kolliphor^®^ 188 Geisman (BASF, Mexico city, México), ethanol, Novag Reglusan™ glibenclamide tablets (5 mg). 

### 2.2. Materials and Equipment

Beaker with magnetic stirrer (Ultraturrax), analytical balance, distillation equipment, drinking trough and cages of acrylic, Malvern Z Sizer-Lab, Mettler Toledo DSC882e, scanning electron microscope (SEM JSM 6010LA, JEOL, Dearborn Road Peabody, MA, USA), Spectra-Pro membrane dialysis tubing 45–50 KDa (Spectra/Por, FL, USA), Labwit ZWY-103D shaker-incubator, ultra-centrifuge, HALO DB-20 UV–Vis double beam spectrophotometer (TechComp, Mexico city, Mexico), and an Accu-chek™ active digital glucometer (Roche, Mexico city, Mexico). 

### 2.3. Animals

Twenty-five Wistar rats (males and females) of 4–5 weeks of age were obtained from the Isolation and Bioterium of the Multidisciplinary Research Unit (UIM) of FES-Cuautitlan-UNAM. Rats were housed under specific conditions in an air-conditioned room and fed standard laboratory food and Milli-Q water ad libitum according to institutional guidelines from the Universidad Nacional Autonoma de Mexico. The experimental procedures were reviewed and finally approved by the Ethics Committee of FES-Iztacala-UNAM (Protocol code CE/FESI/102021/1431 and approval date: 29 October 2021).

### 2.4. Nanoparticle Development

#### 2.4.1. Nanoparticles

Polymeric nanoparticles loaded with glibenclamide were prepared using the solvent evaporation method. This technique was the first to be described for the synthesis of polymeric nanoparticles. The standard procedure involves dissolving the polymer in an organic solvent while the water is present with a stabilizer (surfactant) to form an emulsion [14]. 

##### Aqueous Phase

The aqueous phase consisted of a 4.5% solution of Poloxamer PF-188 as a surfactant.

##### Organic Phase 

A sample of 77 mg of glibenclamide was dissolved in a 1:1 ratio with the Kollicoat MAE 100P polymer and 3 g of Kolliphor RH-40 (co-solvent) in 100 mL of ethanol. The mixture was left to stir until the components were completely dissolved.

##### NP Obtention 

The organic phase was added dropwise to the aqueous phase that was under stirring at 2200 rpm using an Ultraturrax, after which a volume of water corresponding to three times the initial volume was added.

### 2.5. Design of Experiment (Screening and Optimization)

In many experiments, the number of factors that can potentially affect the quality of a process is too great to study all of them in detail [15]. The design of experiments and statical analysis was carried out in two parts using the statical software Statgraphics Centurion XVII (Statgraphics Technologies Inc., VA, USA). The first stage consisted of the screening design, whose objective is to help select an appropriate design including the number of replicates and central points and identify the most important factors that affect the quality of the process, after which the second phase consists of performing an optimization design that provides more information on the relationships between the factors with the greatest impact and the response variables [15]. 

A Plackett–Burman model was used for the screening design. Its elaboration and analysis were carried out by Statgraphics Centurion software (Statgrahics Technologies Inc., VA, USA). Based on the preliminary tests, we selected as factors the drug/polymer ratio, PF-188 concentration, amount of RH-40, stirring speed and time (Table 1), and as responses, size, zeta potential, PDI, and encapsulation efficiency (EE) (Table 2).

In the optimization stage, a central compound design was used: 2^2 starry, which studied the effects of two factors that had significance in the screening process in addition to one other factor thought to affect the process of elaboration of the NPs (Table 3); all were conducted in a single block with 16 runs. The order of the experiments was completely randomized to provide protection against the effects of hidden variables. The expected responses shown in Table 4 are the same as those for the screening phase.

### 2.6. Physicochemical Characterization 

#### 2.6.1. Particle Size, Polydispersity Index (PDI), and Zeta Potential (ζ-Potential)

The determination was based on the dynamic light scattering technique, a non-invasive method that allows us to measure the size and distribution of molecules and particles, typically in the submicron region [16]. Particle size, PDI, and ζ-potential measurements were performed using a particle size analyzer (ZetaSizer Pro, Malvern Instruments, Worcestershire, UK). A volume of 2 mL of NP suspensions were 10-fold diluted with deionized water to reduce scattering and viscosity effects. All samples were analyzed in a disposable capillary cell (DTS1070) at room temperature (25 °C) with an equilibration time of 60 s. Triplicates of each sample were measured and each measurement comprised 10 runs to obtain a stable reading. Results were analyzed using the ZS Xplorer software. 

#### 2.6.2. Drug Release Profiles 

**Figure 1:**
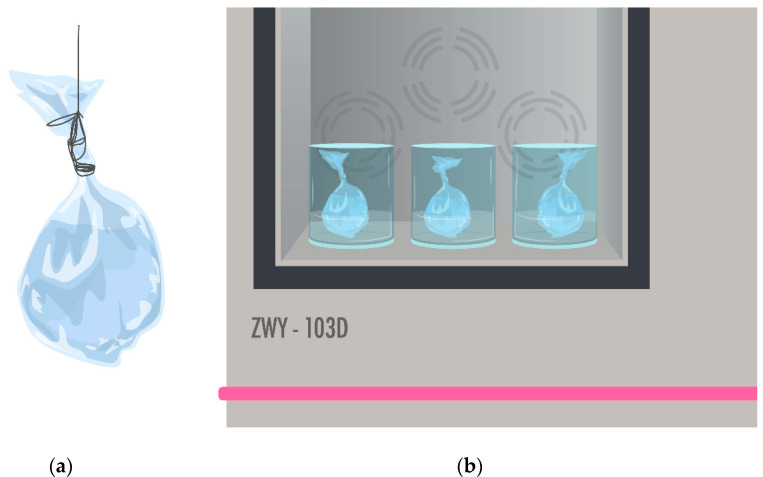
(**a**) Dialysis tubing with MA/EA NP suspension to evaluate glibenclamide release; (**b**) Shaker-incubator with dialysis tubing in PBS pH 7.4 solution.

Once the ME/EA NPs were optimally shaped, a release profile was performed to establish the time it takes to release or cross a semi-permeable barrier to the release medium. A total of 100 mL of NP suspension was taken and deposited in dialysis tubing with a pore size of 45–50 kDa. The dialysis tubing was placed in 500 mL of solution PBS pH 7.4, introducing the system in a shaker incubator at 37 °C with a constant stirring of 5 rpm (Figure 1). Samples with a volume of 3 mL were collected at 5, 10, 20, 40, 60 min and later at 2, 4, 6, 8, 24, 48, and 72 h. Quantification of glibenclamide was made using a HALO DB-20 UV–Vis double beam spectrophotometer.

The method was validated using the validation guide for analytical methods published by the National College of Pharmaceutical Chemists and Biologist A.C. [17].

#### 2.6.3. Scanning Electronic Microscopy 

Morphology and size were determined for optimal formulation using scanning electron microscopy (SEM; JSM 6010LA, JEOL, Dearborn Road Peabody, MA, USA). Before introducing the sample, it was coated with ionized gold with the sputtering technique under the following conditions of sputter 7 Amps 300 s, which allowed us to observe the NP sample under microscopy. 

#### 2.6.4. Encapsulation Efficiency 

**Figure 2:**
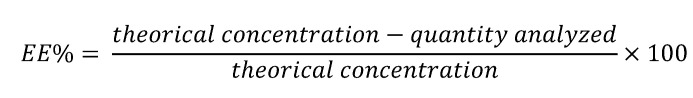
Encapsulation efficiency equation.

Encapsulation efficiency was determined by indirect method. Accordingly, a 3 mL aliquot of the NP suspension was taken and placed in 1.5 mL Eppendorf^®^ tubes, subsequently ultracentrifuged at 14,000 rpm for 45 min, and the resulting supernatant was quantified for the non-encapsulated glibenclamide by UV–Vis spectrophotometry at a wavelength of 305 nm. The concentration of encapsulated glibenclamide was determined by the difference of concentration obtained in the supernatant and the theoretical concentration added in the NPs, thus obtaining the actual concentration present in the NPs, as shown in Figure 2.

The method was validated using the validation guide for analytical methods published by the National College of Pharmaceutical Chemists and Biologist A.C [17].

### 2.7. In Vivo Model

An animal model for biomedical research is one in which normative biology or behavior can be studied, or in which a spontaneous or inducing pathological process can be investigated, and in which the phenomenon in one or more respects resembles the same phenomenon in humans or others species of animals [18].

Wistar strain rats of 4–5 weeks of age and approximately 200 g weight were selected from the Isolation and Bioterium of the Multidisciplinary Research Unit of the FES Cuautitlan-UNAM. The groups were assembled using a Japanese snake curve arranging the animals in order of weight from smallest to largest, and the groups can be seen in Table 5.

**Figure 3:**
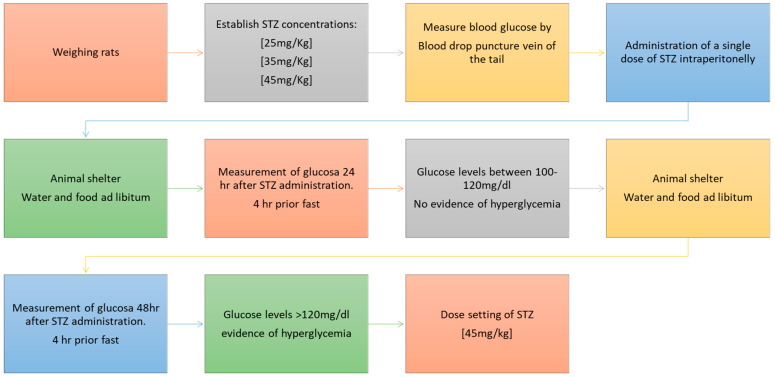
Selection of the dose of STZ to generate hyperglycemia in rats.

For the induction of diabetes in the animal model, a previous study was carried out where different concentrations of streptozotocin (STZ) (25 mg/Kg, 35 mg/kg, and 45 mg/kg) were administered in a single dose, after which it was established that the effective dose for the development of diabetes was 45 mg/kg (Figure 3). Once the effective concentration (45 mg/kg) was established, a single-dose of STZ in citrate buffer (0.05 M) was administered intraperitoneally to Wistar rats according to the protocol established by Barriga-Gutiérrez [19,20].

The blood samples were taken by puncture of the vein located at the base of the tail and the determination of glucose level was carried out with an Accu-chek™ Active digital glucometer using reactive strips. 

**Figure 4:**
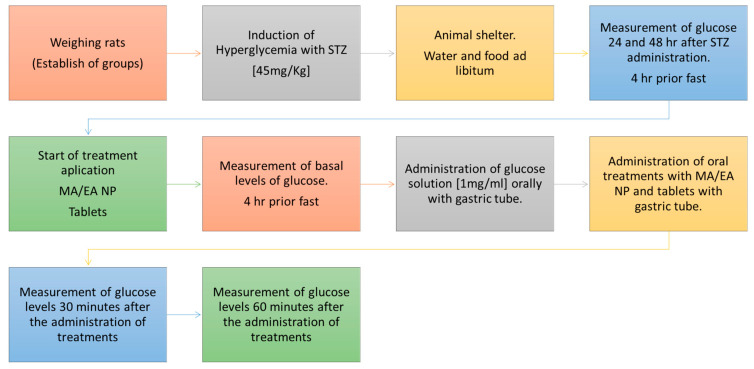
Application of treatments and determination of blood glucose levels.

Before sampling, the animals fasted for 4 h and those with glucose levels ≥120 mg/dL were considered diabetic. During the experiment, the animals were orally administered with a glucose solution at a concentration of 1 g/mL and immediately afterwad with 1 mL of NP suspension (0.9 mg of glibenclamide) or with the Novag Reglusan™ 5 mg glibenclamide tablet using a gastric tube. The tablet was pulverized and resuspended in Milli-Q water, then loaded into a syringe and administered orally. Two more samples were taken, the first at 30 min and the second at 60 min (Figure 4). 

## 3. Results

### 3.1. Results and Discussion 

#### 3.1.1. Screening Design 

Polymeric nanoparticles loaded with glibenclamide were designed and characterized using the solvent evaporation method. In the screening phase, a Plackett–Burman design was established that helped us to detect the factors that were important during the synthesis of NPs (Table 6). 

**Figure 5:**
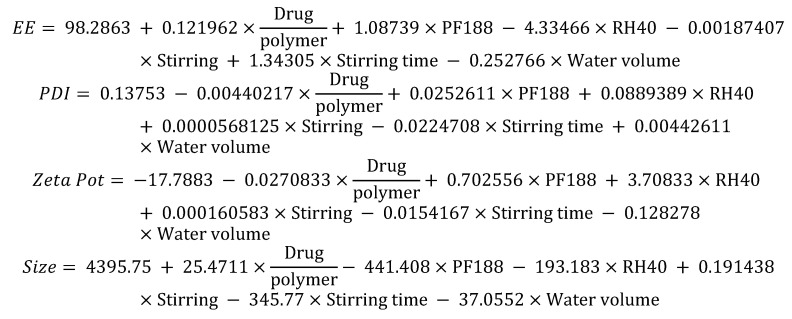
Equations showing the interactions of the different factors in the development of NPs in the screening phase.

A total of 15 experiments were carried out with six factors and four responses, the interaction of which is represented in the equations shown in Figure 5.

**Figure 6:**
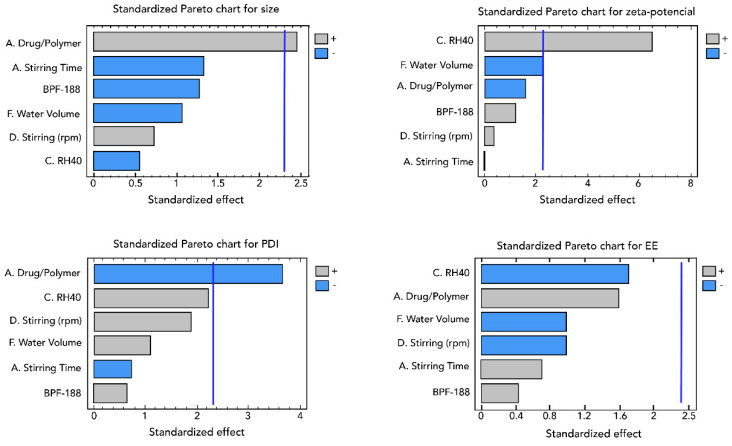
Pareto diagrams showing the factors with significant response in the screening design for the synthesis of NPs with glibenclamide.

When analyzing the responses obtained in the established design, it was determined that only two factors were significant in the responses when developing glibenclamide NPs. These results can be corroborated in the Pareto diagrams (Figure 6), pointing out that the drug/polymer and RH-40 ratios are the factors that significantly impact the formulation of NPs.

Screening experiments are effective in identifying significant variables by making the process as economic as possible. This could be achieved by carefully choosing the size of the experiment and the combination set of input variables (the design) to be executed in the experiment [21]. Variables that were determined as significant from the screening experiment (drug/polymer ratio and Kolliphor RH40) were further investigated in an optimization design. 

#### 3.1.2. Optimization of Nanoparticles

Through the screening design, we were able to determine that the factors drug/polymer and Kolliphor RH-40^®^ were those that presented significance in the response. For this reason, a new optimization model was developed, this time only with these two variables (Table 7).

**Figure 7:**
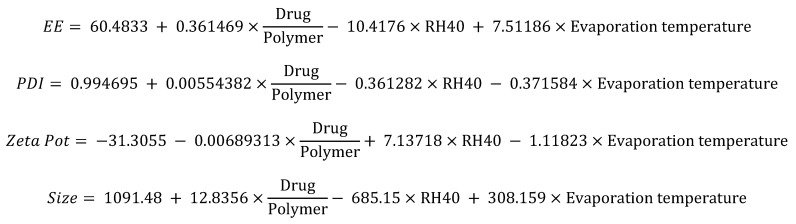
Interaction between factors for any response in the optimization phase.

A star-like central compound response surface model was established that will allow us to analyze the effects of the drug/polymer and Kolliphor RH-40^®^ relationship, and based on this, determine the formulation with the best physicochemical characteristics. Moreover, we added another factor that we thought could affect the selected responses. This factor was the evaporation temperature. A total of 16 experiments were conducted with six factors and four responses, whose interactions are represented in the equations shown in Figure 7.

**Figure 8:**
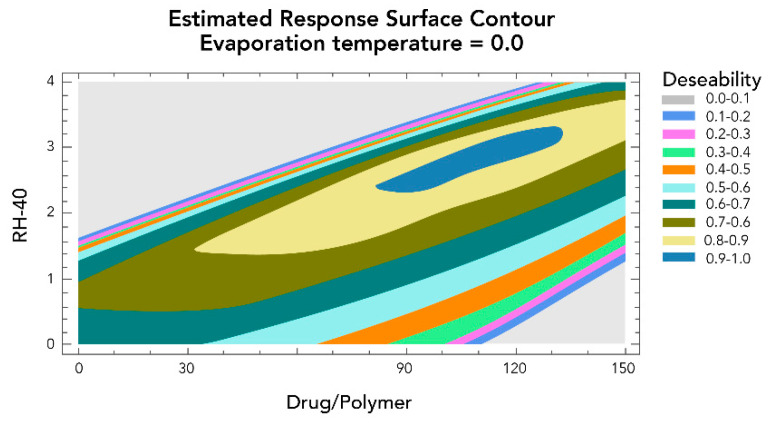
Response surface plot for the development of nanoparticles of 50 nm in diameter.

Based on the response surface graphs obtained after the optimization test (Figure 8), we decided to perform the physicochemical characterization tests.

#### 3.1.3. Size, PDI, and Zeta Potential

Zeta potential is the electric potential that exist in the particle cut plane, with a short distance from the surface. Colloidal particles dispersed in a solution are electrically charged thanks to their ionic characteristics and bipolar characteristics. 

The principle of determining zeta potential is simple: a controlled electric field is applied by means of the electrodes immersed in a sample in suspension; this caused the charged particles to move across the electrode of opposite polarity. The viscous forces act on the movement, establishing a balance between both electrostatic attractive forces and the viscosity resistance [22].

The Brownian movement of the particles or molecules in suspension causes the laser light to be scattered in different intensities. 

**Figure 9:**
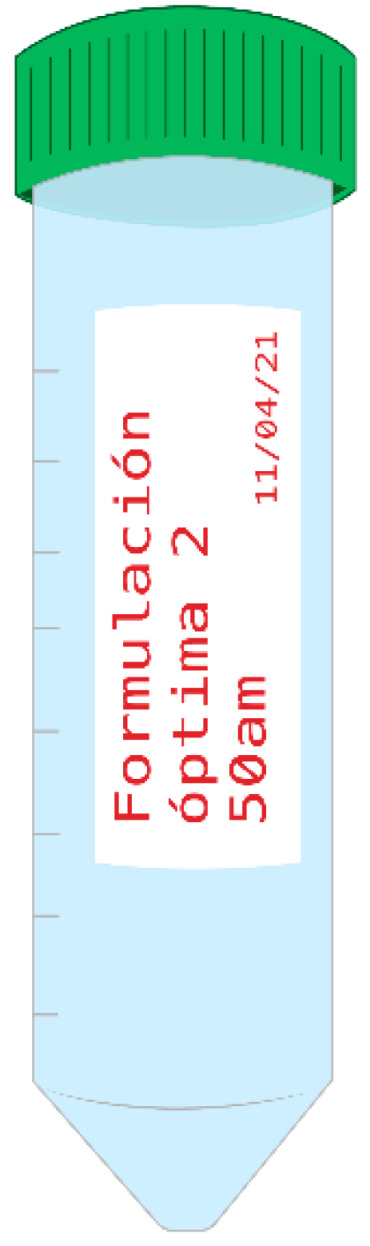
Formulation of optimized glibenclamide NPs.

The physical characteristics (PDI, zeta potential, and size) of the optimal formulation proposed (Figure 9) were evaluated in triplicate in a Zetasizer Malvern equipment. The results obtained are shown in Table 8.

#### 3.1.4. Encapsulation Efficiency 

**Figure 10:**
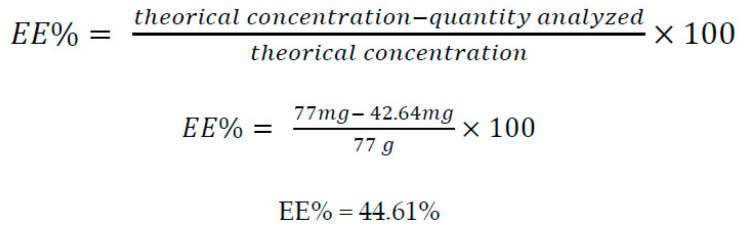
Equation substituted with units to obtain the encapsulation efficiency.

This was determined by dividing the actual concentration of previously quantified encapsulated glibenclamide by the theoretical amount of glibenclamide in the NPs (Figure 10). After the triplicate quantification, the results obtained are shown in Table 9.

#### 3.1.5. Scanning Electronic Microscopy (SEM)

Scanning electron microscopy (SEM) is one of the most versatile instruments available for the examination of microstructure morphology and chemical composition characterization [23]. 

Because of their nanometric size, NPs are impossible to observe with the naked eyes, so we use SEM that will allow us to amplify our sample with high resolution when passing an electron beam through it, for this purpose.

**Figure 11:**
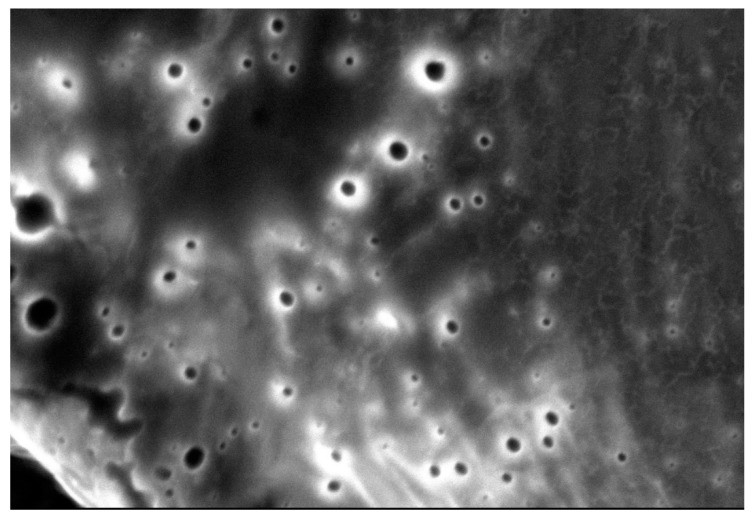
SEM of the optimized formulation of MA/EA copolymer NPs loaded with glibenclamide, size 19nm magnification 2500×.

To observe the shape and corroborate the size obtained in the Zetasizer Malvern equipment, scanning electron microscopy (SEM) was performed. In Figure 11, the images obtained by the SEM are shown, exhibiting the spherical morphology of the methacrylic acid–ethyl acrylate copolymer nanoparticles loaded with glibenclamide in addition to their sizes, which were similar to those obtained in previous tests. 

#### 3.1.6. Drug Release Profiles and Kinetic Drug Release

The dissolution of a drug is a prerequisite for the absorption and clinical response of most oral drugs. The in vitro release of a drug from the pharmaceutical form containing it depends on the physicochemical characteristics of the drug, the excipients used, and the technology used for the manufacture [24,25]. There are different kinetic models that describe the release of drugs from their pharmaceutical forms as the qualitative and quantitative changes in a formulation can alter the release of the drug, and therefore, its performance in vivo. The use of in vitro drug dissolution data to produce in vivo bio performance should be considered as the rational development of a modified release formulation. These models are based on different mathematical functions that describe the dissolution profile. These include the zero-order model, the first-order model, the Higuchi model, and the Korsmeyer–Peppas model, among others [26].

Today, the copolymer of methacrylic acid and ethyl acrylate is widely used for the preparation of pH-sensitive NPs. To precisely control the release of drugs, NPs prepared with Eudragit^®^ generally mixed with other polymers are expected to serve as a platform for the oral administration of hydrophilic or hydrophobic macromolecules, pharmaceutical active peptides/proteins, glycosaminoglycans, and oligonucleotides [27]. 

**Figure 12:**
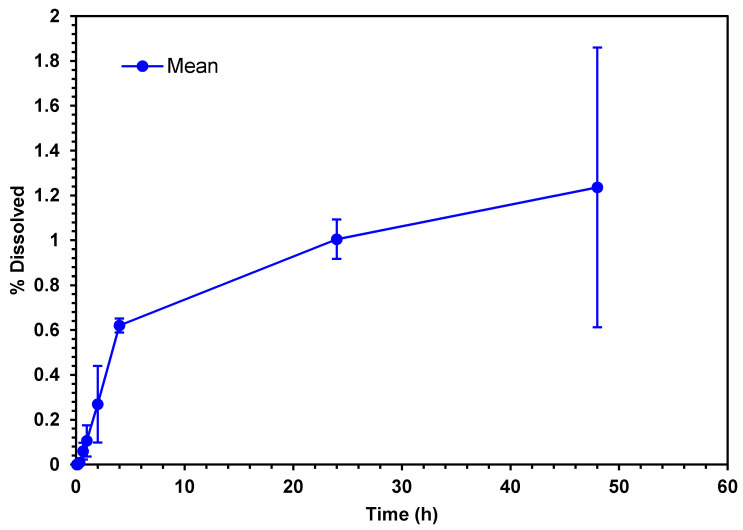
Drug release profiles of the optimized formulation of MA/EA NPs in dialysis tubing bags.

The MA/EA NPs developed showed strong pH-dependent drug release properties in acidic and neutral pH values followed by a sustained release at pH 7.4 [28]. The result of the release profile (Figure 12) showed that the release of glibenclamide lasted up to 50 h in a PBS solution of pH 7.4 after the administration of the MA/EA NPs. This corresponds to what is described in the literature and with the characteristics of Eudragit^®^ polymers, regarding the advantage of pH-sensitive MA/EA NPs over non-sensitive NPs, where most carriers used are approved for enteric administration as the rapid dissolution and /or swelling at specific pH results in quick drug release and a high drug concentration gradient, and the bio-adhesion to mucosal becomes high, which can facilitate drug absorption [27,28].

**Figure 13:**
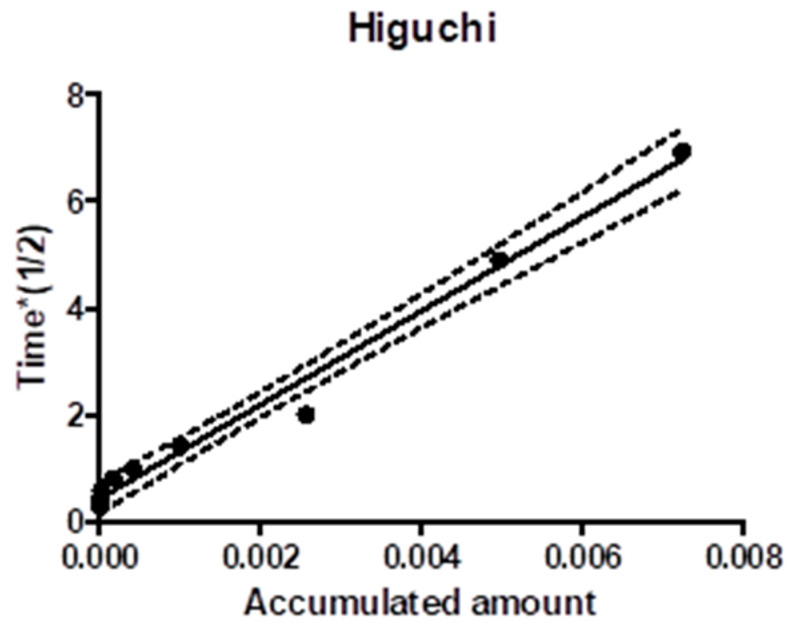
Higuchi model where r^2^ = 0.9850.

After the analysis of the drug release profiles of the MA/EA NPs, it was determined that the release model to which it best fit was Higuchi’s when obtaining an r^2^ = 0.9850 (Figure 13), based on the hypothesis that drug diffusion occurs in only one dimension, that the dissolution and swelling of the matrix are negligible, that the diffusivity of the drug is constant, and that perfect sink conditions are always reached in the solution medium [26].

#### 3.1.7. In Vivo Tests

Diabetes was induced using streptozotocin (STZ), a compound that has been shown to be cytotoxic to pancreatic beta cells. STZ is a compound of glucosamine-nitrosourea derived from the fungus *Streptomyces achromogenes* and is usually used as a chemotherapeutic agent in the treatment of pancreatic beta cell carcinoma. STZ induces one type of diabetes that is like diabetes mellitus with non-ketosis hyperglycemia in some animals species [29,30,31].

STZ can induce diabetes through two routes depending on the dose. It poses high selectivity by pancreatic beta cells because of their structural similarity to glucose, allowing the STZ to bind to the glucose transporter receptor GLUT2 by accumulating in these cells by initiating an autoimmune process that results in the destruction of the Langerhans islets beta cells with the onset of clinical diabetes within 24–72 h [29,30]. 

**Figure 14:**
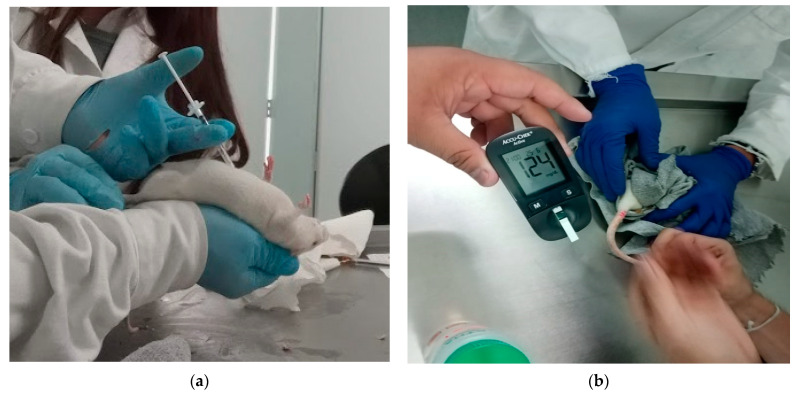
(**a**) STZ administration through the intraperitoneal. (**b**) Glucose levels greater that 120 mg/dL are indicative of animals with diabetes.

After 48 h after administration of STZ (Figure 14), glucose levels were evaluated according to the protocol established by Gutierrez-Barriga, showing values above 120 mg/dL of glucose in blood, establishing in this way that the animals suffered from diabetes [20].

**Figure 15:**
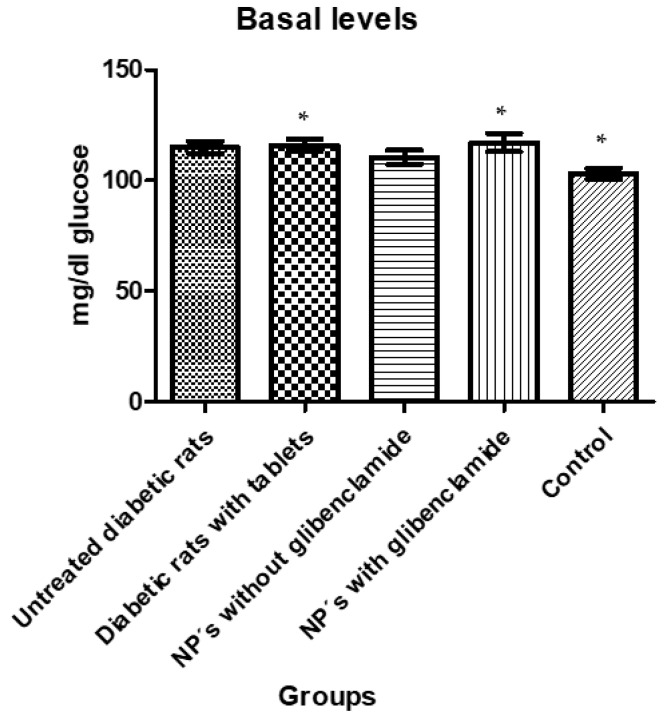
Baselines glucose levels showing significant differences between groups. The asterisks (*) represent a statistically significant difference between groups (*p* < 0.05).

A statically significant difference (*p* < 0.05) between the groups with diabetic rats and the control group was observed (Figure 15). From Figure 13 as well as from Table 10, it emerges that only groups 2 and 4 showed significantly higher levels than the control group, that is, group 5 of non-diabetics.

To corroborate the significant difference obtained in the analysis of variance (ANOVA), a Tukey test (Table 10) was performed in which we could also establish the differences between the groups.

**Figure 16:**
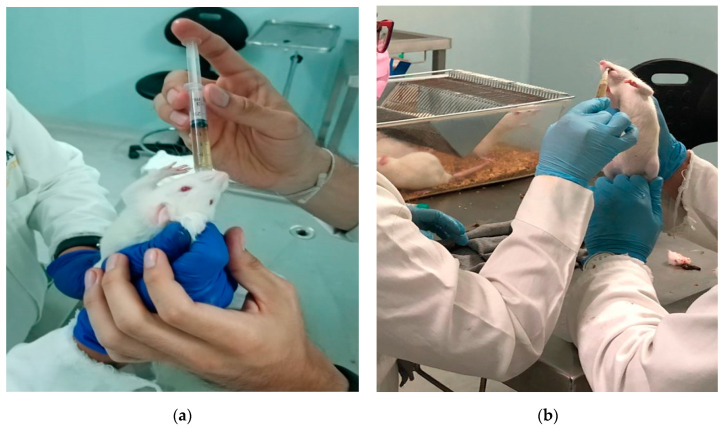
(**a**) Oral administration of glucose solution (1 g/mL). (**b**) Oral administration of MA/EA NPs suspension.

**Figure 17:**
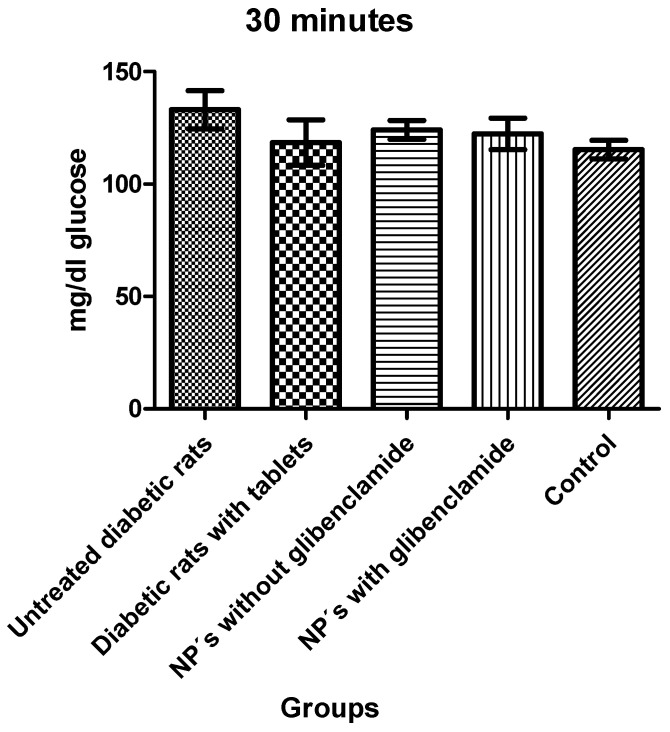
Glucose levels 30 min after administration of a glucose solution and glibenclamide treatments.

After the intake of basal glucose, the animals were administered with a glucose solution (1 g/mL), to trigger their glucose levels and to be able to observe more accurately the glucose changes between the different groups and their treatments (Figure 16). The first intake after administration of both glucose and treatments was performed at 30 min (Figure 17) where an increase in serum glucose concentrations was observed in all groups. After performing a statical analysis (ANOVA), it showed that in this period, there was no significant difference between any of the five groups.

**Figure 18:**
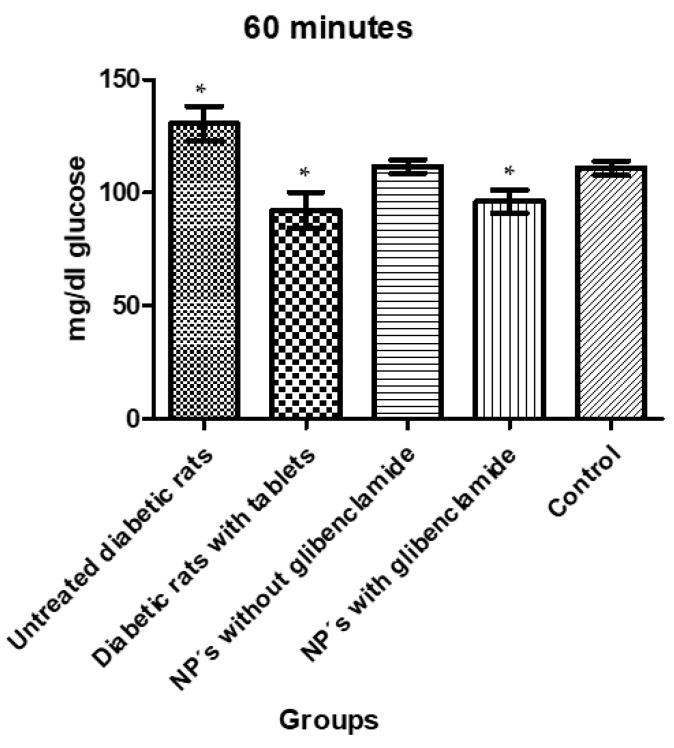
Glucose levels 60 min after administration of a glucose solution and glibenclamide treatments. The asterisks (*) represent a statistically significant difference between groups (*p* < 0.05).

The third and last intake was made after 60 min in which the glucose levels were maintained or a decrease in the control and treatment groups could be observed. These changes can be seen in Figure 18, which show a significant difference between group 1 with untreated diabetic animals compared to groups 2 and 4 administered with commercial tablets and MA/EA NPs, respectively. 

The administration of MA/EA NPs loaded with glibenclamide (0.9 mg) was shown to be effective by decreasing glucose levels in animals administered; significant differences between the treated and untreated groups were achieved by obtaining a *p* > 0.05. This was corroborated by performing a Tukey test (Table 11) showing the difference between the group with untreated diabetic rats (group 1), group 2, and group 4.

It is important to note that with a lower dose of glibenclamide administered with NPs, it is possible to achieve a significant effect in lowering glucose values even when they are approximately 5-fold lower than conventional administration with tablets. The foregoing highlights the characteristics of the use of NPs as drug carriers by improving the bioavailability of the drug.

The decrease in glucose levels in the group of animals administered with MA/EA NPs without glibenclamide (group 3) can be explained by the metabolic variety that exists between the different animals, although all the animals presented hyperglycemia (glucose > 120 mg/dL). The glucose values between them do not necessarily have to be the same, as this behavior resembles the metabolic diversity existing among the human population with diabetes, so this effect is not associated with the administration of the MA/EA NPs in such a way that it is not statistically significant.

## 4. Conclusions

Novel NPs based on a methacrylic acid–ethyl acrylate copolymer loaded with glibenclamide were developed through screening and optimization design. It was determined that the factors that significantly impacted on the characterization were the drug/polymer ratio and amount of Kolliphor-RH40^®^ obtained at the end of NPs with the physicochemical characteristics best suited to serve as an alternative vehicle for the administration of glibenclamide as treatment for diabetes mellitus type 2. Furthermore, administering NPs to an animal model that previously developed diabetes proved to be effective in reducing blood glucose levels with a significant difference against untreated animals, even with a 5-fold lower glibenclamide concentration and compared to the evaluated commercial form Novag Reglusan™ glibenclamide tablets.

## Figures and Tables

**Table 1:** Independent variables and levels selected for the screening design.

Factors	Low	High	Units
Drug/polymer	50	150	Mg
PF-188	1.5	4.5	%
RH-40	1.5	4.5	G
Stirring speed	2000	6000	Rpm
Stirring time	2.0	6.0	Min
Volume of water	45	75	mL

**Table 2:** Dependent variables selected in the optimization phase.

Response	Units
Size	Nm
Zeta potential	mV
PDI	--
Encapsulation efficiency (EE)	%

**Table 3:** Independent variables in the optimization of glibenclamide ME/EA NPs.

Factors	Low	High	Units	Continuous
Drug/Polymer	40	120	mg	Yes
RH-40	1	3	g	Yes
Evaporation temperature	−1	1	°C	Yes

**Table 4:** Dependent variables in the optimization phase.

Response	Units
Size	Nm
Zeta potential	mV
PDI	--
EE	%

**Table 5:** Group in which the 25 rats were classified by weight order.

Animals Were Divided into 5 Different Groups with 5 Individuals Each as Follows:
Group 1: untreated diabetic rats
Group 2: diabetic rats with conventional pharmaceutical form (tablets)
Group 3: diabetic rats administered with NP without glibenclamide
Group 4: diabetic rats administered with NP with glibenclamide
Group 5: control

**Table 6:** Factors and responses in the screening phase.

Drug/Polymer	PF-188	Kolliphor RH-40^®^	Stirring	Stirring Time	Water Volume	Size	Zeta Pot	PDI	EE
mg	%	G	rpm	min	mL	Nm	mV		%
50	4.5	4.5	6000	2	75	24.59	−11.42	1.2	75.253666
150	1.5	4.5	6000	2	75	4687	−10.14	0.2939	63.397299
150	4.5	1.5	6000	6	45	3891	−16.22	0.1027	99.312885
50	1.5	1.5	6000	6	75	1634	−23.44	0.4802	87.46687
150	1.5	4.5	2000	2	45	5117	−11.54	0.189	94.337278
150	4.5	1.5	6000	2	45	3664	−20.68	0.2228	98.847868
100	3	3	4000	4	60	5136	−10.69	0.3943	97.082929
150	4.5	4.5	2000	6	75	14.79	−11.54	0.4279	88.191434
150	1.5	1.5	2000	6	75	1893	−27	0.0299	101.33701
50	1.5	1.5	2000	2	45	2245	−18.03	0.3975	90.442499
50	4.5	1.5	2000	2	75	37.11	−17	0.5533	70.439951
100	3	3	4000	4	60	4577	−14.36	0.7203	74.129591
50	1.5	4.5	6000	6	45	22.13	−5.168	0.969	62.067704
100	3	3	4000	4	60	12.74	−12.66	0.3544	62.876815
50	4.5	4.5	2000	6	45	21.3	−5.812	0.3075	86.575837

**Table 7:** Factors and responses established for optimization design.

Block	Drug/Polymer	Kolliphor RH-40^®^	Evaporation Temperature	Size	Zeta Potential	PDI	EE
	Mg	G	°C	nm	mV		%
1	40	1	1	1565	−18.19	0.6814	79.491841
1	80	2	1.68179	756	−18.93	0.6576	83.9436238
1	120	1	−1	1823	−24.94	4.418	93.0083276
1	80	3.68179	0	13.97	−13.35	0.2083	76.8001864
1	147.272	2	0	1250	−17.75	0.3323	80.6717907
1	120	1	1	3979	−25.41	0.04271	94.2851601
1	80	2	0	34.43	−13.67	0.3513	47.9191043
1	40	1	−1	32.4	−23.82	1.374	79.6281078
1	120	3	−1	746	0.02799	0.9255	88.8181854
1	120	3	1	22.02	−13.71	0.4585	87.1761386
1	80	2	−1.68179	15.83	−15.17	0.3648	10.3197805
1	80	2	0	27.75	−18.33	0.08396	63.2713165
1	40	3	−1	15.93	−12.29	0.3544	43.862202
1	12.7283	2	0	19.16	−13.77	0.3824	44.8243383
1	40	3	1	14.97	−12.66	0.3222	23.1320136
1	80	0.318207	0	1653	−39.36	0.4928	99.8984444

**Table 8:** Size, PDI, and zeta potential of the optimal MA/EA NP formulation.

Expected Size	Size (nm)	PDI	Zeta Potential
50 nm	18.98 +/− 9.14	0.37085 +/− 0.014	−13.7125 +/− 1.82

**Table 9:** Load capacity and encapsulation efficiency of methacrylic acid–ethyl acrylate copolymer nanoparticles.

Absorbance 305 nm	Dilution	Quantification (µg/mL)	Dilution Factor	Quantification (mg)	Theoretical Load (mg)	Load Capacity (mg)	Encapsulation Efficiency (%)
1.4907 1.4865 1.4907	(1:2)	251.118644	502.237288	42.6901695	77	34.3098305	44.5582214
	(1:2)	250.40678	500.813559	42.5691525	77	34.4308475	44.7153863
	(1:2)	251.118644	502.237288	42.6901695	77	34.3098305	44.5582214
1.4893	(1:2)	250.881356	501.762712	42.6498305	77	34.3501695	44.61 +/- 0.22

**Table 10:** Tukey test showing the groups that showed a statistically significant difference in baseline levels.

Tukey’s Multiple Comparison Test	Significant *p* < 0.05	Summary	95% CI of diff
Group 1 vs. Group 2	No	ns	−13.78 to 12.00
Group 1 vs. Group 3	No	ns	−8.380 to 17.40
Group 1 vs. Group 4	No	ns	−15.03 to 10.75
Group 1 vs. Group 5	No	ns	−0.9797 to 24.80
Group 2 vs. Group 3	No	ns	−6.755 to 17.55
Group 2 vs. Group 4	No	ns	−13.40 to 10.90
Group 2 vs. Group 5	Yes	*	0.6451 to 24.95
Group 3 vs. Group 4	No	ns	−18.80 to 5.505
Group 3 vs. Group 5	No	ns	−4.755 to 19.55
Group 4 vs. Group 5	Yes	*	1.895 to 26.20

ns: non-significant. * significant (*p* < 0.05).

**Table 11:** Tukey test showing the groups that showed a statistically significant difference at 60 min.

Tukey’s Multiple Comparison Test	Significant *p* < 0.05	Summary	95% CI of diff
Group 1 vs. Group 2	Yes	***	15.42 to 61.18
Group 1 vs. Group 3	No	ns	−4.005 to 41.76
Group 1 vs. Group 4	Yes	***	11.52 to 57.29
Group 1 vs. Group 5	No	ns	−3.008 to 42.23
Group 2 vs. Group 3	No	ns	−41.30 to 2.460
Group 2 vs. Group 4	No	ns	−25.78 to 17.99
Group 2 vs. Group 5	No	ns	−40.29 to 2.919
Group 3 vs. Group 4	No	ns	−6.354 to 37.41
Group 3 vs. Group 5	No	ns	−20.87 to 22.34
Group 4 vs. Group 5	No	ns	−36.40 to 6.813

ns: non-significant. *** significant (*p* < 0.05).

## Abbreviations

PDI: polidispersion index, DM: diabetes mellitus, DM2: type 2 diabetes mellitus, NPs: nanoparticles, NP: nanoparticle, EE: encapsulation efficiency, SEM: scanning electron microscopy, MA/EA NPs: methacrylic acid–ethyl acrylate copolymer nanoparticles, STZ: streptozotocin, ANOVA: analysis of variance.

## References

[1] Conget D.I. (2002). Diagnosis, classification and pathogenesis of diabetes mellitus. Rev. Esp. Cardiol..

[2] Sanamé F.A.R., Álvarez M.L.P., Figueredo E.A., Estupiñan M.R., Rizo Y.J. (2016). Tratamiento Actual de la Diabetes Mellitus Tipo 2. Correo Científico Médico.

[3] Cuartero C., Glibenclamida C., Tabletas E.N. (2005). Validación de un método analítico espectrofotométrico para cuantificar glibenclamida en tabletas de 5 mg Validation of spectrophotometric analytical method to quantify glibenclamide. Rev. Mex. De Cienc. Farm..

[4] Luzi L., Pozza G. (1997). Glibenclamide: An old drug with a novel mechanism of action?. Acta Diabetol..

[5] Devarajan P.V., Sonavane G.S. (2007). Preparation and in vitro/in vivo evaluation of gliclazide loaded Eudragit nanoparticles as a sustained release carriers. Drug Dev. Ind. Pharm..

[6] Sola D., Rossi L., Schianca G.P.C., Maffioli P., Bigliocca M., Mella R., Corlianò F., Paolo Fra G., Bartoli E., Derosa G. (2015). Sulfonylureas and their use in clinical practice. Arch. Med. Sci..

[7] Winkler G., Gerő L. (2011). Pharmacogenetics of insulin secretagogue antidiabetics. Orv. Hetil..

[8] Tessier D., Dawson K., Tetrault J.P., Bravo G., Meneilly G.S. (1994). Glibenclamide vs gliclazide in type 2 diabetes of the elderly. Diabet. Med..

[9] Holstein A., Plaschke A., Egberts E.H. (2001). Lower incidence of severe hypoglycaemia in patients with type 2 diabetes treated with glimepiride versus glibenclamide. Diabetes. Metab. Res. Rev..

[10] Chamundeeswari M., Jeslin J., Verma M.L. (2019). Nanocarriers for drug delivery applications. Environ. Chem. Lett..

[11] Mendes A.N., Hubber I., Siqueira M., Moreno Barbosa G., De D., Moreira L., Holandino C., Pinto J.J., Nele M. Preparation and Cytotoxicity of Poly(Methyl Methacrylate) Nanoparticles for Drug Encapsulation. www.ms-journal.de.

[12] Dupeyrón D., Rieumont J., González M., Castaño V.M., Dupeyr on D., Gonz alez M., Casta V.M. (2009). Protein Delivery by Enteric Copolymer Nanoparticles. J. Dispers. Sci. Technol..

[13] Urrejola M.C., Soto L.V., Zumarán C.C., Peñaloza J.P., Álvarez B., Fuentevilla I., Haidar Z.S. (2018). Sistemas de Nanopartículas Poliméricas II: Estructura, Métodos de Elaboración, Características, Propiedades, Biofuncionalización y Tecnologías de Auto-Ensamblaje Capa por Capa (Layer-by-Layer Self-Assembly). Int. J. Morphol..

[14] Lu Y., Shah K.W., Xu J. (2017). Synthesis, morphologies and building applications of nanostructured polymers. Polymers.

[15] Diseños de Cribado—Minitab. https://support.minitab.com/es-mx/minitab/19/help-and-how-to/statistical-modeling/doe/supporting-topics/factorial-and-screening-designs/screening-designs/.

[16] D Dispersión de luz dinámica DLS|Malvern Panalytical. https://www.malvernpanalytical.com/es/products/technology/light-scattering/dynamic-light-scattering.

[17] García M.A., Soberón E., Cortés M., Rodríguez R., HJ A.A. (2002). Guía de Validación de Métodos Analíticos.

[18] Chatzigeorgiou A., Halapas A., Kalafatakis K., Kamper E. (2009). The Use of Animal Models in the Study of Diabetes Mellitus. In Vivo.

[19] Like A.A., Rossini A.A. (1976). Streptozotocin-induced pancreatic insulitis: New model of diabetes mellitus. Science.

[20] Gutierrez Barriga S.A. (2020). Efecto de la Suplementación con Tributirina vía Oral en Ratas con Diabetes e Insuficiencia Renal. Ph.D. Thesis.

[21] Woods D.C., Lewis S.M., Ghanem R., Higdon D., Owhadi H. (2017). Design of Experiments for Screening. Handbook of Uncertainty Quantification.

[22] Potencial Zeta—Lenntech. https://www.lenntech.es/potential-zeta.htm.

[23] Zhou W., Apkarian R., Wang Z.L., Joy D. (2007). Fundamentals of scanning electron microscopy (SEM). Scanning Microscopy for Nanotechnology.

[24] Cook H.J., de Anda Jáuregui G., Carrasco K.R., Cruz L.M. (2012). Comparación de Perfiles de Disolución: Impacto de los Criterios de Diferentes Agencias Regulatorias en el Cálculo de ƒ2. http://www.scielo.org.mx/scielo.php?script=sci_arttext&pid=S1870-01952012000300007.

[25] Castañeda P.S., Olvera L.G., Bernad M.J.B., López H.S., Escobar-Chávez J.J. (2021). Development of a Spectrophotometric Method for the Determination of Florfenicol in Eudragit Nanocapsules. Pharm. Chem. J..

[26] Minotta J. Evaluación de la Cinetica de Liberacion de un Fármaco Modelo con Clasificación Biofarmaceutica Clase II, Desde Matrices Comprimidas Compuestas por Materiales Polimericos Anionicos. https://repository.icesi.edu.co/biblioteca_digital/bitstream/10906/83047/1/jimenez_farmaco_biofarmaceutica_2017.pdf.

[27] Sonaje K., Lin K.-J., Wang J.-J., Mi F.-L., Chen C.-T., Juang J.-H., Sung H.-W. (2010). Self-Assembled pH-Sensitive Nanoparticles: A Platform for Oral Delivery of Protein Drugs. Adv. Funct. Mater..

[28] Wang X.Q., Zhang Q. (2012). pH-sensitive polymeric nanoparticles to improve oral bioavailability of peptide/protein drugs and poorly water-soluble drugs. Eur. J. Pharm. Biopharm..

[29] Graham M.L., Janecek J.L., Kittredge J.A., Hering B.J., Schuurman H.J. (2011). The streptozotocin-induced diabetic nude mouse model: Differences between animals from different sources. Comp. Med..

[30] Akbarzadeh A., Norouzian D., Mehrabi M., Jamshidi S., Farhangi A., Allah Verdi A., Mofidian S., Lame Rad B., Akbarzadeh A. (2007). Induction of diabetes by streptozotocin in rats. Indian J. Clin. Biochem..

[31] Ikebukuro K., Adachi Y., Yamada Y., Fujimoto S., Seino Y., Oyaizu H., Hioki K., Ikehara S. (2002). Treatment of streptozotocin-induced diabetes mellitus by transplantation of islet cells plus bone marrow cells via portal vein in rats. Transplantation.

